# An Essential Role of the Cytoplasmic Tail of CXCR4 in G-Protein Signaling and Organogenesis

**DOI:** 10.1371/journal.pone.0015397

**Published:** 2010-11-19

**Authors:** Darran G. Cronshaw, Yuchun Nie, Janelle Waite, Yong-Rui Zou

**Affiliations:** Center for Autoimmune and Musculoskeletal Diseases, Feinstein Institute for Medical Research, Manhasset, New York, United States of America; New York University, United States of America

## Abstract

CXCR4 regulates cell proliferation, enhances cell survival and induces chemotaxis, yet molecular mechanisms underlying its signaling remain elusive. Like all other G-protein coupled receptors (GPCRs), CXCR4 delivers signals through G-protein-dependent and -independent pathways, the latter involving its serine-rich cytoplasmic tail. To evaluate the signaling and biological contribution of this G-protein-independent pathway, we generated mutant mice that express cytoplasmic tail-truncated CXCR4 (ΔT) by a gene knock-in approach. We found that ΔT mice exhibited multiple developmental defects, with not only G-protein-independent but also G-protein-dependent signaling events completely abolished, despite ΔT's ability to still associate with G-proteins. These results reveal an essential positive regulatory role of the cytoplasmic tail in CXCR4 signaling and suggest the tail is crucial for mediating G-protein activation and initiating crosstalk between G-protein-dependent and G-protein-independent pathways for correct GPCR signaling.

## Introduction

CXCR4 is a seven-transmembrane GPCR for the chemokine, CXCL12. Both CXCR4 and CXCL12 are broadly expressed by cells of multiple tissues and play an indispensable role in embryogenesis [1]. Genetic ablation of CXCR4 or CXCL12 leads to embryonic lethality, as a result of defects in cardiogenesis, vascular development, hematopoiesis, and the CNS [2]–[6]. In adulthood, CXCR4 and CXCL12 have been implicated in pathogenesis of autoimmune diseases and tumor metastasis [7]–[10]. However, the precise molecular mechanisms that underlie these diverse physiological and pathological functions remain obscure.

Like the majority of GPCRs, CXCR4 contains a highly conserved DRY motif (Asp-Arg-Tyr) located in the second intracellular loop. Extensive studies using rhodopsin and adrenergic receptors as models have established a general paradigm for GPCR activation. It proposes that ligation of GPCR triggers protonation of the Asp residue in the DRY motif, inducing conformational changes of the GPCR and activation of the interacting G proteins [11], [12]. Mutation of the DRY motif of chemokine receptors prevents ligand-induced activation of the pertussis toxin (PTX)-sensitive Gαi proteins and abolishes generation of second messengers and chemotaxis, indicating a pivotal role of the DRY motif in G-protein mediated signaling [13]–[16]. Increasing evidence shows GPCRs may also exert biological effects independent of G-protein function. The C-terminal (CT) tail of GPCRs is rich in serines and threonines, and truncation of the tail of several chemokine receptors abrogates ligand-activated receptor phosphorylation, demonstrating that the tail of these receptors is the only phosphorylation target of GPCR kinases (GRKs) [17], [18]. Phosphorylated GPCR tail binds to β-arrestins, leading to rapid internalization and desensitization of the ligand-activated receptor [19], [20]. In addition to mediating receptor internalization, β-arrestins also serve as scaffold proteins, recruiting Src family tyrosine kinases to the phosphorylated GPCRs and consequently activate MAP kinases [21]. Given that GPCRs may deliver signals through the DRY motif and its cytoplasmic tail, it is important to determine whether the DRY motif and the tail of CXCR4 act as independent signaling transduction modules that carry out distinct cellular functions.

The functional importance of the CT tail of CXCR4 has been underscored by identification of truncating mutations of CXCR4 in patients with WHIM (warts, hypogammaglobulinemia, immunodeficiency, and myelokathexis) syndrome. WHIM patients carry autosomal dominant mutations in *Cxcr4* that eliminate a part of the serine-rich CT tail [22]. Considerable studies have been conducted using mutant cells from WHIM patients or a variety of cell lines transfected with truncational mutants of CXCR4 to investigate WHIM pathogenesis. While all these data show that deletion of the tail impairs ligand induced receptor internalization, the biochemical and cellular responses, however, seem to be highly variable in these *in vitro* systems, with variations in MAPK activation and chemotaxis [23]–[28]. These discrepancies could be attributed to different expression levels of the transgenic CXCR4 as well as to different signaling machinery available in the utilized cell lines. To overcome these problems, we generated mutant mice that express tail-truncated CXCR4 by a “knock-in” approach and used these mice to investigate the developmental, cellular, and biochemical functions of the CXCR4 tail under physiological conditions. Results of the present study reveal that truncation of the CT tail of CXCR4 not only obliterates G-protein independent signaling pathways mediated by tail-associated factors, but also prevents signaling through Gαi, resulting in similar developmental defects as seen in CXCR4-null mice.

## Results

### Generation of CXCR4-ΔT mice

**Figure 1:**
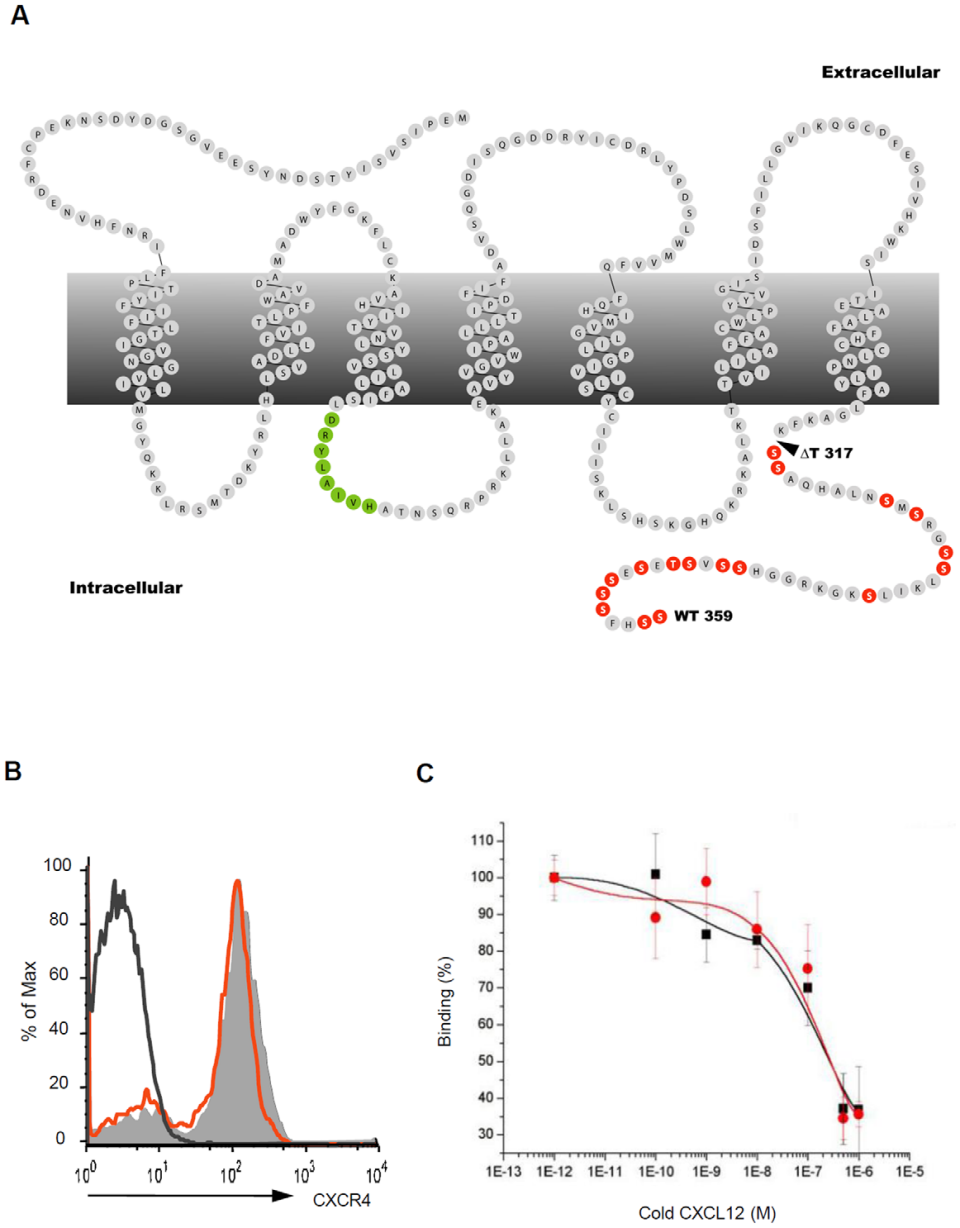
Tail-truncated CXCR4 is able to bind CXCL12. (*A*) Snake-plot of mCXCR4 highlights two signaling modules, the DRY motif in green and the serine and threonine residues in the CT tail of CXCR4 in orange. The arrowhead indicates the site of truncation in ΔT mice. (*B*) Histograms show the expression of CXCR4 on WT (shaded histogram) or ΔT FL cells (red line) isolated from E18.5 embryos. The histogram with black line represents isotype control. (*C*) Percentages of ^125^I-CXCL12 bound to HEK cells expressing WT CXCR4 (black curve) or ΔT (red curve) in the presence of increasing concentrations of unlabelled CXCL12 (n = 3, mean ± SD).

The cytoplasmic tail of CXCR4 contains 16 serine residues which are the putative targets of GRKs. In order to evaluate the precise biological functions mediated by the CT tail of CXCR4 we removed the last 42 amino acids from CXCR4 (aa 318–359), thereby completely eliminating the sequences that confer GRK activity and β-arrestin recruitment (Fig. 1*A*, mutant CXCR4 is hereafter denoted as ΔT). To ensure a physiological expression of ΔT, we generated “knock-in” mice (Fig. S1). Our results showed that ΔT was expressed at an identical level to that of wildtype (WT) CXCR4 on myeloid cells in embryonic day (E) 18.5 fetal liver (Fig. 1*B*). Furthermore, ΔT bound CXCL12 with an affinity similar to that of WT CXCR4 (Fig. 1*C*). In this report, we refer to the mutant mice carrying the CXCR4 tail-truncated allele as “ΔT mice”.

### ΔT mutants phenocopy the developmental defects of CXCR4-null mice

Previous reports have shown that CXCR4-null mice exhibit multiple developmental defects and die perinatally [3], [4], [29]. Such an embryonic lethal phenotype was not rescued in ΔT mice, however, unlike CXCR4-null mice of which only 50% survived till E17.5 and all with reduced body sizes [4], ΔT embryos were present at Mendelian ratios till E18.5 with grossly normal body sizes and morphology (data not shown).

**Figure 2:**
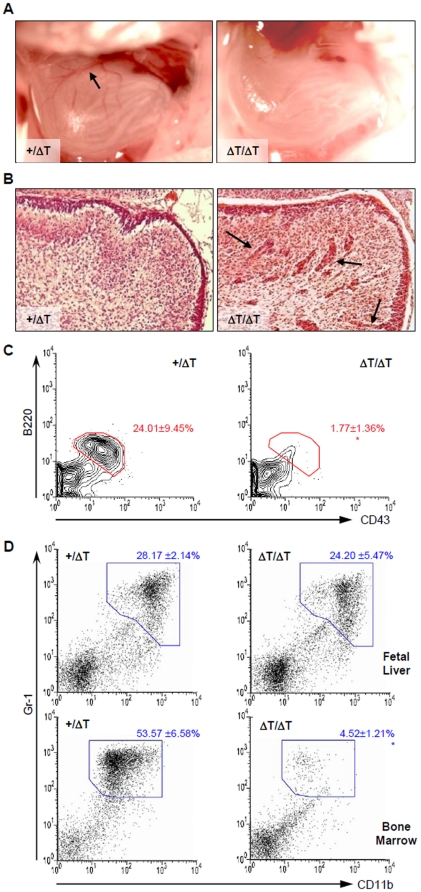
ΔT mice display identical developmental defects to those in CXCR4 null mice. (*A*) Gross morphology of the E18.5 stomach of WT and ΔT mice demonstrates that large vessels (arrow) were absent in ΔT mice. (*B*) Hematoxylin and eosin-stained sagittal sections of the E18.5 cerebellums show dislocated EGL cells (arrows) in mutant mice. (*C*) Contour plots reveal a reduced compartment of the B220^+^CD43^+^ pro-B cells in the E18.5 ΔT FL. Numbers indicate percentages of cells within the indicated gate (mean ± SD, n = 7. *, P<0.0001, calculated by the two-tailed Student's *t* test). (*D*) Dot plots show that generation of myeloid lineage cells (Gr1^+^CD11b^+^) is normal in the FL but deficient in BM of E18.5 embryos. Values are mean ± the SD (n = 3).

To determine to what extent the ΔT mutation affected organogenesis, we examined those organs of which formation was critically dependent on CXCR4. We found that, similar to that observed in CXCR4-null mice, ΔT mice lacked large vessels in the gastrointestinal area (Fig. 2*A*); had displaced cells of the external granule layer (EGL) in the cerebellum (Fig. 2*B*); defective B cell genesis (Fig. 2*C*) and severely impaired bone marrow (BM) myelopoiesis (Fig. 2*D*). These results together indicate that, although the overall impact on animal development is relatively milder, the ΔT mutation imposes almost identical effects as the CXCR4-null mutation on the development of neural, vascular and hematopoietic systems.

Since ΔT mice were embryonic lethal, we generated CXCR4^f/ΔT^ mice and crossed them to transgenic CD19-Cre (B cell specific) and ROSA^ET2-CRE^ mice (tamoxifen induced) to delete the floxed *Cxcr4* allele and conditionally express ΔT in specific lineages of cells. We also established pro-B cell lines by Abelson virus transformation for *in vitro* studies to determine cellular and biochemical functions of the CXCR4 tail.

### The CXCR4 tail is critical for CXCL12-induced chemotaxis but dispensable for integrin-mediated adhesion

**Figure 3:**
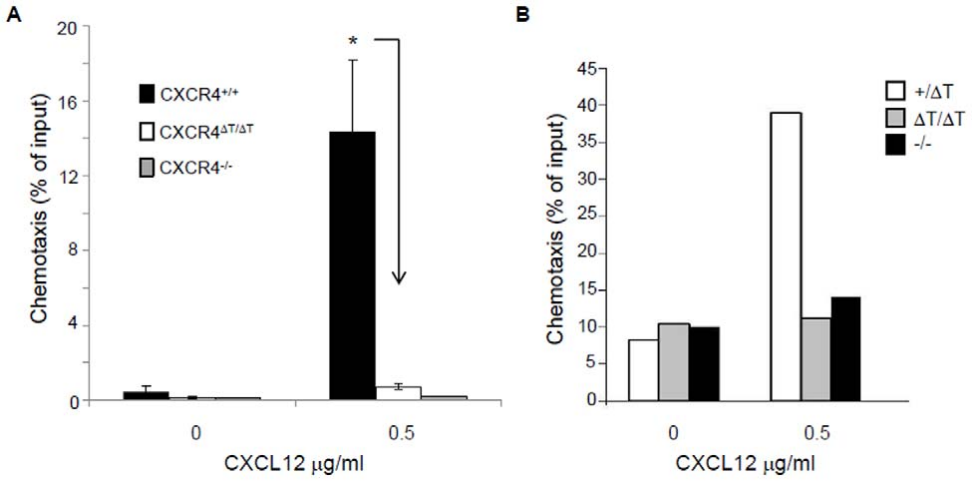
CXCR4-mediated chemotaxis is abolished in mutant cells. (*A*) Transwell-assay measurement of CXCL12-induced chemotaxis of WT, CXCR4-null and ΔT pro-B cells. Results obtained from three independent experiments are indicated as percent of total input. (*, P<0.0005, calculated by the two-tailed Student's *t* test). (*B*) E18.5 FL cells from WT, CXCR4-null and ΔT mice were subjected to CXCL12-induced chemotaxis. The migration response is from a single experiment representative of at least three groups of embryos.

At the late gestation, the primary site of hematopoiesis shifts from the fetal liver (FL) to BM. This process is dependent on CXCR4-mediated migration of hematopoietic stem cells (HSCs) [2], [4], [29]. Since myelopoiesis was relatively normal in the FL but defective in BM of ΔT mice (Fig. 2*D*), it was conceivable that the ΔT mutation impaired BM homing of hematopoietic cells. To determine whether the CXCR4 tail was required for chemotaxis, we measured the chemotactic response of ΔT pro-B cells toward CXCL12 by a transwell assay. We found that while 15–20% of WT pro-B cells underwent chemotaxis toward CXCL12, less than 2% of ΔT cells migrated under the same condition (Fig. 3*A*). Abolishment of CXCL12-induced chemotaxis was also found for the FL hematopoietic cells (Fig. 3*B*), demonstrating that the CXCR4 tail is essential for CXCL12-mediated chemotaxis.

We have shown previously that CXCR4 signaling promotes retention of B-cell precursors in the BM [30], presumably through VCAM-1/VLA-4 mediated firm adhesion [31], [32] and lack of CXCR4 leads to the release of these precursors into the periphery.

**Figure 4:**
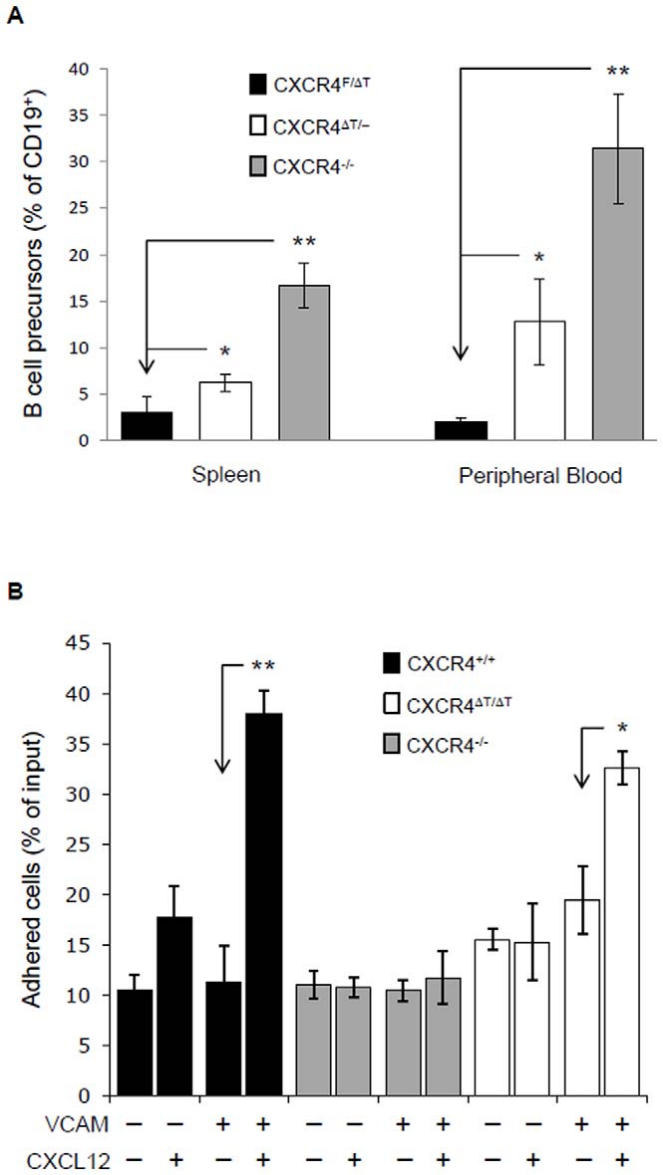
ΔT largely restores the defective BM retention and cell adhesion of CXCR4-null B-lineage precursors. (*A*) Peripheral blood cells and splenocytes were isolated from 6 wk old WT, CD19-Cre+ CXCR4^f/f^ and CD19-Cre+ CXCR4^f/ΔT^ mice, and stained with anti-CD19, B220 and IgM. CD19^+^-gated cells can be further distinguished as B-cell precursors (B220^lo^IgM^−^) and mature B cell (B220^hi^IgM^+^) populations. Bar graphs show percentages of B-cell precursors within the total CD19^+^ B-cell population detected in the periphery. Results were obtained from 5–8 mice of each group (*, P<0.001; **, P<0.000005, calculated by the two-tailed Student's *t* test). (*B*) CXCL12-stimulated WT, CXCR4-null and ΔT pro-B cells were analyzed for their ability to adhere to VCAM-1. Data are displayed as mean ± SD from three independent experiments (*, P<0.001; **, P<0.0005, calculated by the two-tailed Student's *t* test).

To examine whether ΔT is still able to facilitate the retention of B-cell precursors in the BM, we analyzed B-cell subsets in peripheral blood and spleen of CD19-Cre- CXCR4^f/ΔT^ (WT), CD19-Cre+ CXCR4^f/ΔT^ (ΔT), and CD19-Cre+ CXCR4^f/f^ mice (CXCR4 null) by flow cytometry. In WT animals, B cell precursors (B220^lo^IgM^−^) were barely detectable in PB (Fig. S2). When *Cxcr4* was deleted, a substantial amount of B cell precursors were released from the BM to the periphery (Fig. 4*A*) and this population was significantly reduced in the in the blood and spleen of ΔT mice, suggesting that ΔT partially restored BM retention (Fig. 4*A* and Fig. S2).

To directly examine whether the cytoplasmic tail of CXCR4 was required for triggering integrin-mediated cell adhesion, we decided to test *in vitro* the ability of ΔT to promote B cell adherence to VCAM-1. To exclude the possible interference from CXCR7, the other receptor for CXCL12, we used ΔT, CXCR4-null and WT pro-B cells in a static adhesion assay. These pro-B cells have an identical cell-surface level of VLA-4 (receptor for VCAM-1) but do not express CXCR7 (Fig. S3). As shown in Fig. 4*B*, WT pro-B cells exhibited a 4-fold increase in VCAM-1 adhesion after CXCL12 stimulation, whereas CXCR4-null cells exhibited only basal VCAM-1-binidng activity. Notably, CXCL12 induced adherence of ΔT cells to VCAM-1 was comparable to that of WT (Fig. 4*B*). These results indicate that tail-truncated CXCR4 is still able to mediate inside-out signaling that activates VLA-4. Interestingly, the adhesion of WT and ΔT mutant pro-B cells to VCAM-1 was not affected by PTX pretreatment (Fig. S4), suggesting that activation of VLA-4 was independent of Gαi signals.

### CT tail deletion abolishes β-arrestin binding and impairs CXCR4 internalization

**Figure 5:**
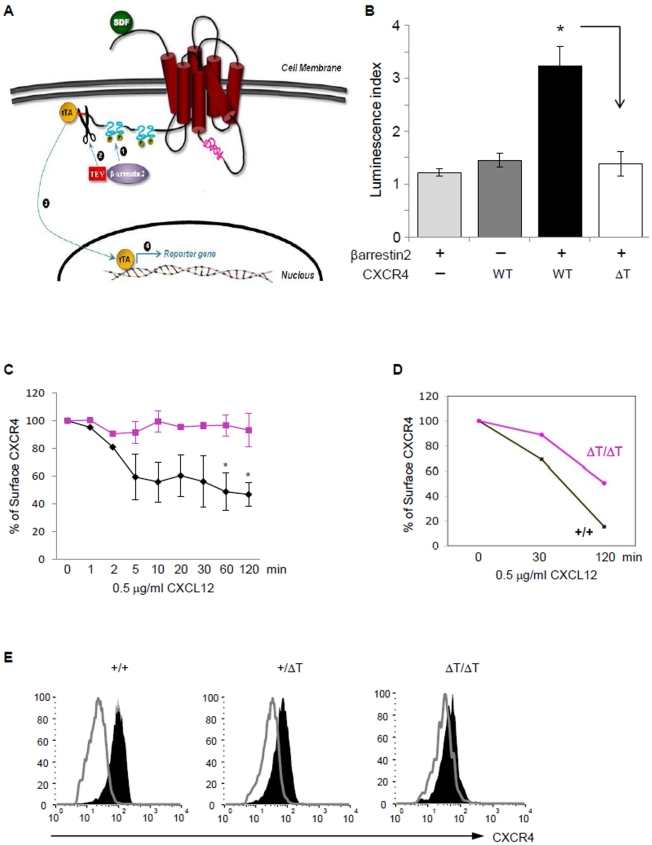
β-arrestin recruitment and receptor internalization are impaired in ΔT cells. (*A*) Schematic diagram illustrates the Tango assay. CXCL12 (SDF) binding to CXCR4 stimulates recruitment of β-arrestin-TEV protease fusion protein (1). The TEV protease cleaves a recognition sequence (highlighted in red) that has been fused, along with the transcriprion factor tTA, to the CT end of CXCR4 (2). This triggers the release of tTA (3) allowing it to enter the nucleus and activate the expression of the tTA-dependent luciferase reporter gene (4). (*B*) HEK cells were transiently transfected with the luciferase reporter vector together with a combination of β-arrestin-TEV, WTCXCR4-tTA, or ΔTCXCR4-tTA as indicated. Cells were stimulated with CXCL12 and measured for luciferase activity. Results from 3 independent experiments are displayed as the luminescence index calculated by the fold increases in luciferase activity in cells ± CXCL12 (*, P<0.01, calculated using the two-tailed Students *t* test). (*C*) The kinetics of receptor downmodulation was determined by measuring cell-surface expression of WT CXCR4 or ΔT on pro-B cells after CXCL12 stimulation for indicated times. Results are from 3 independent experiments (*, P<0.05, calculated using the two-tailed Student's *t* test). (*D*) CXCL12-induced receptor internalization on E18.5 FL cells was carried out as described in (*C*) above. (*E*) Representative histograms show CXCR4 expression on FL cells of WT, heterozygous and homozygous ΔT mice with (thick line) or without (shaded) 2 h CXCL12 incubation.

Whether the tail of CXCR4 is the only cytoplasmic domain that recruits β-arrestins remains controversial. Whereas a number of studies have shown that CXCR4 is primarily associated with β-arrestins through its CT tail [27], [33], others can co-IP tail-truncated CXCR4 with β-arrestins [26], [34] or detect it co-localized with β-arrestins by microscopy [25]. To detect the interaction between β-arrestins and ΔT, we performed a Tango assay [35] in which ligand-induced rapid and transient interaction between CXCR4 and β-arrestins is converted into an accumulative transcriptional response (Fig. 5*A*). We found that reporter cells expressing WTCXCR4-tTA exhibited an increased luciferase activity after CXCL12 stimulation. In contrast, such CXCL12-induced luciferase gene activation was not detected in ΔTCXCR4-tTA-expressing reporter cells (Fig. 5*B*). This provides strong evidence that association between CXCR4 and β-arrestin2 absolutely depends on its cytoplasmic tail.

Interaction of β-arrestins and the phosphorylated CT tail of GPCRs has been shown to cause agonist-induced receptor internalization, a process considered as an important step in signal desensitization [36]. As shown in Fig. 5*C*, for pro-B cells, CXCL12 treatment induced internalization of 35–40% of cell surface WT CXCR4 within 5 min, and maximal internalization of 50% was achieved at 60 min. On the contrary, CXCL12 binding to ΔT did not trigger endocytosis at all. Interestingly, FL cells downmodulated surface WT CXCR4 in response to CXCL12 with slower kinetics but stronger magnitude compared to that in pro-B cells. Similar to that seen in mutant pro-B cells, there was no obvious internalization of ΔT in FL cells 30 min after CXCL12 stimulation. However, after 2 h of CXCL12 incubation ∼50% of ΔT had been internalized in FL cells compared to 80% for WT (Fig. 5*D*). Heterozygous ΔT mutant cells displayed an intermediate level of receptor internalization (Fig. 5*E*), a phenotype previously seen in cells from some patients with WHIM syndrome [23]. Taken together, our data indicate that the truncation of the CXCR4 tail severely compromises CXCL12-induced receptor internalization in primary cells. The disparity in results observed between FL and pro-B cells could be explained by FL cells expressing CXCR7 that can form heterodimers with CXCR4 [37]. Therefore, internalization of ligand-bound CXCR7 could result in partial downmodulation of heterodimerized ΔT, a process that would not occur in pro-B cells which do not express CXCR7.

### The CT tail is critical for CXCR4 G-protein-dependent and -independent signaling

It remains to be determined whether signals initiated from G proteins and the cytoplasmic tail-associated molecules may act independently or must cross-talk to coordinately regulate proper cellular functions. Therefore, we decided to use our ΔT mutant cells to dissect the role of the CXCR4 tail in the CXCR4 signaling cascade.

**Figure 6:**
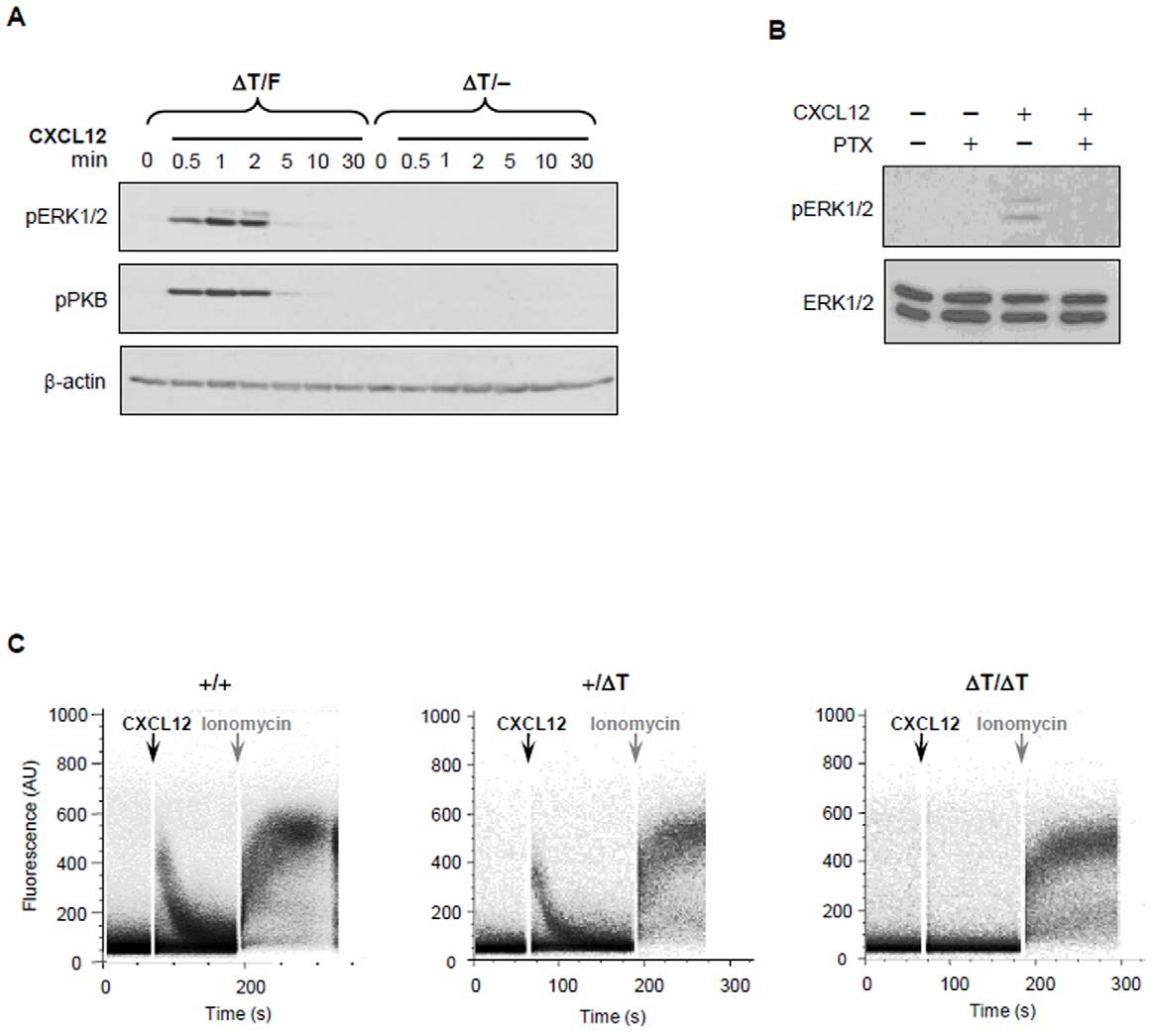
Impaired signaling pathways in ΔT cells. (*A*) Splenocytes from conditional ΔT mice and their WT littermates were stimulated with 0.5 µg/ml CXCL12 for indicated times and immunoblot analysis for phosphorylated PKB at serine 473 (pPKB) or phosphorylated ERK (pERK) were performed. Equal loading of protein was confirmed by anti-β-actin staining. Results are representative of at least 3 independent experiments. (*B*) WT splenocytes, pretreated with 100 ng/ml of PTX for 1 h, were stimulated with 0.5 µg/ml of CXCL12 for 1 min and lysates immunoblotted for pERK and re-probed with antibody against total ERK1/2 for loading control. (*C*) FL cells from WT, heterozygous and homozygous ΔT embryos were collected and loaded with Fluo-4 and Fura-red calcium indicator dyes. Cells were stimulated with 0.5 µg/ml CXCL12 for 2 min followed by 2 µM ionomycin. Results are representative of 3 independent experiments.

To obtain sufficient primary ΔT cells for biochemical studies, we generated ROSA^ET2-CRE^ CXCR4-ΔT/F mice in which the floxed *Cxcr4* gene (*Cxcr4^F^*) can be deleted by tamoxifen induced Cre, allowing only ΔT expression in adult tissues (referred to as conditional ΔT mice). Treatment of the conditional ΔT mice with tamoxifen led to nearly complete deletion of the *Cxcr4^F^* allele in splenocytes, and the overall lymphoid and myeloid compartments of the spleen in the mutant and WT mice were comparable (data not shown). To determine whether the ΔT mutation impaired CXCR4 signaling, we first examined activation of ERK and PKB, which have been shown to be downstream of both β-arrestin and G protein signaling pathways [38]. We found that both ERK and PKB were rapidly and transiently phosphorylated in WT cells after CXCL12 stimulation (Fig. 6*A*). This signaling event was dependent on Gαi protein activation, as PTX treatment blocked their phosphorylation completely (Fig. 6*B*). To our surprise, stimulation of ΔT splenocytes with CXCL12 failed to induce measurable levels of phosphorylation of either ERK or PKB, suggesting that the lack of the CXCR4 tail impairs G-protein activation (Fig. 6*A*). Similar results were also obtained using FL cells and Abelson transformed pro-B cells (Fig. S5), indicating that the observed defect in the activation of these signaling pathways is a ubiquitous rather than a cell-lineage specific phenomenon.

To further evaluate whether the tail contributed to CXCR4 mediated G-protein signaling, we examined CXCL12 induced intracellular calcium mobilization, a key signaling event known to be activated only by Gβγ [39]. Surprisingly, we found that CXCL12 stimulation was not able to elicit calcium flux in ΔT cells (Fig. 6*C*). These results suggest that the CXCR4 tail may play an essential role in G-protein activation.

### CXCR4 mediated Gαi protein activation is dependent on the CXCR4 tail

**Figure 7:**
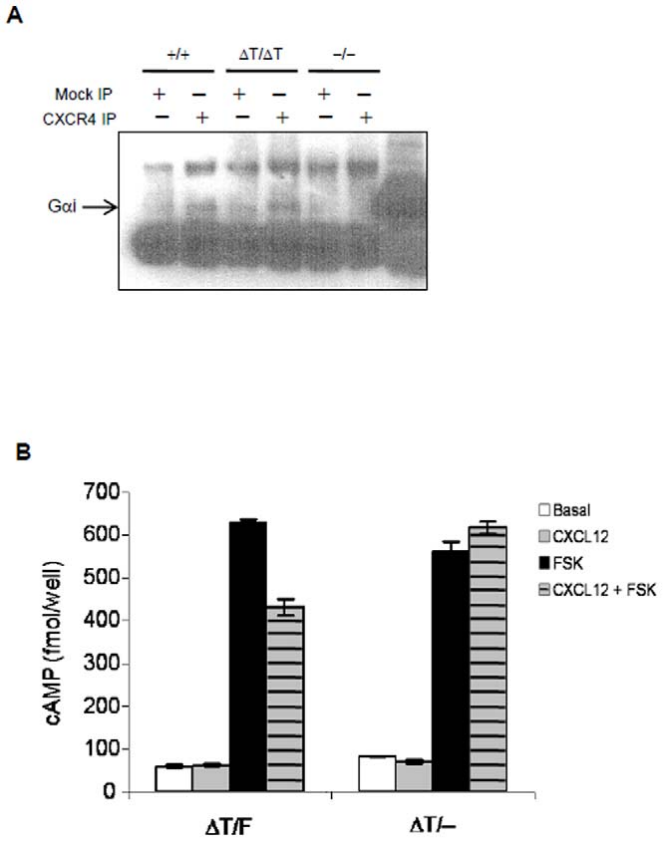
The CXCR4 tail is essential for G-protein activation but not association. (*A*) WT and ΔT pro-B cell lysates were immunoprecipitated with anti-CXCR4 antibody and immunoblotted for Gαi. (*B*) Splenocytes from WT and conditional ΔT mice were used for assessment of Gαi activities measured by CXCL12-induced inhibition of intracellular cAMP production. The cAMP levels in untreated cells (open bars), in cells stimulated with 1 µg/ml of CXCL12 for 10 min (gray bars), in cells pretreated with 1 µM forskolin (FSK, black bars) and in cells treated with FSK and CXCL12 (hatched bars) were determined by ELISA.

Mutational studies showed that a cluster of amino acid residues located at the N-terminal of the cytoplasmic tail of GPCRs was critical for G-protein activation [40]. However, it is not clear whether the CT tail was actually involved in G-protein coupling. Since our data showed that ΔT failed to activate multiple signaling pathways downstream of G proteins, we sought to determine whether the ΔT mutation altered the physical interaction between CXCR4 and heterotrimeric G proteins. We found that anti-CXCR4 immunoprecipitated an equivalent amount of Gαi in WT and ΔT cells (Fig. 7*A*), indicating that heterotrimeric G-proteins are still physically associated with ΔT.

Although the CXCR4 tail was not required for G-protein association, it may control G-protein activity. To assess this possibility, we examined Gαi activity by measuring intracellular cAMP levels after CXCL12 stimulation. In WT cells, CXCL12-activated Gαi proteins inhibited forskolin-induced adenylyl cyclase activity, leading to the suppression in intracellular cAMP production. However, the forskolin elevated intracellular cAMP level was not attenuated in ΔT cells after CXCL12 stimulation (Fig. 7*B*). These data demonstrate that the CXCR4 tail, although dispensable for G-protein association, is required for heterotrimeric G-protein activation.

## Discussion

Models regarding GPCR activation and signaling have been rapidly modified to accommodate new data derived from structural and mutational analysis of GPCRs. In a classical model, GPCRs mediate virtually all signaling events through their coupled G proteins, and the cytoplasmic tail of GPCRs plays only a negative regulatory role through the interacting β-arrestins to downmodulate ligand-activated receptors and desensitize cells from further ligand stimulation [41]. This view has been changed after the finding of “ligand bias”, namely, a GPCR engaged to different ligands can activate G proteins without inducing receptor internalization or can activate downstream MAPK pathways through β-arrestins in the absence of detectable G protein activities [42]–[44]. In a revised model, different agonists may induce the same GPCR to assume different active states, resulting in exposure of distinct cytoplasmic interfaces to different interacting molecules (the G proteins or β-arrestins) that contribute to various cellular outcomes [45].

The physiological significance of these signaling mechanisms has not been delineated in vivo. Here, we used CXCR4 as a model molecule to elucidate the cellular effects mediated by individual signaling modules of GPCRs. For this reason, we generated ΔT mice. Our results demonstrate that the CXCR4 tail plays an essential function of CXCR4 in the developing brain, vascular and hematopoietic systems. However, the embryonic lethality of CXCR4-ΔT mice was unexpected, as patients with WHIM syndrome who also harbor truncating mutations at the CXCR4 C-terminus exhibited a dominant gain of function phenotype. We speculate that C-terminus truncated CXCR4 in WHIM patients might lose desensitization function but retain partial signaling properties through the remaining 8 to 12 serine residues in its tail. This postulation is supported by recent findings indicating that site-specific phosphorylation of serine residues at the CXCR4 tail is regulated by different GRKs which have either positive or negative impact on CXCR4 signaling [33]. Further studies are required to elucidate whether knock-in mice expressing the same mutant form of CXCR4 as WHIM patients recapitulate the human pathogenesis.

A question that remains unanswered is how the CXCR4 tail facilitates G-protein activation. G proteins are presumably activated through a cascade of conformational changes upon ligand binding to GPCRs. Due to the high flexibility of the C-terminus of GPCRs, this portion has been deleted to promote crystallization of GPCRs for structural studies [46], [47]. Therefore, no information can be inferred from the available structures on the interdependence between the C-terminus of GPCRs and GPCR-associated G proteins. Based on biochemical studies, the tail of GPCRs is known to interact with GRKs and β-arrestins. Although it is unclear whether such an interaction facilitates G-protein activation, experiments from β-arrestin2 and GRK6 knock-out mice seem to suggest otherwise [48]. It is conceivable that the tail itself or other unknown associated molecules may help to facilitate the conformational change and activation of GPCR interacting heterotrimeric G proteins upon ligand stimulation [49]. Further mutagenesis and proteomics studies may help to resolve this issue.

It should be mentioned that ΔT embryos exhibited a relatively milder developmental retardation as compared to the CXCR4-null mice. Consistently, the ΔT mutation completely abolishes the chemotaxis function but still retained the adhesion ability of WT pro-B cells to VCAM-1 upon CXCL12 stimulation. These results thus reveal the existence of a CT tail and G-protein independent pathway responsible for CXCR4-induced inside-out signaling for integrin activation. In agreement with this notion, it has been shown that activation of CXCR7, results in integrin-mediated adhesion without evoking obvious G-protein signaling [50]. In the case of CXCR4, we and others have found that PTX could completely block CXCL12 induced chemotaxis but not adhesion [51]. At present, the nature of this tail- and G protein-independent pathway remains unclear.

In summary, we generated ΔT mice to directly determine the signaling properties of the CXCR4 tail and to assess the physiological relevance of this signaling module. Results from our studies have revealed an unexpected regulatory role of the tail in G-protein activation. This discovery is important not only for understanding the mechanisms of GPCR activation and function but is also pertinent for future drug development.

## Materials and Methods

### Ethics Statement

All experiments were reviewed and approved by the Institutional Animal Care and Use Committee at the Feinstein Institute for Medical Research (IACUC Protocol #2009-023).

### Mice

Strategies for gene targeting, ES cell screening and genotyping of ΔT mice are provided in *Fig. S1*. To generate mice that express ΔT in adult tissues, ΔT mice were bred to previously described ROSA^ET2-CRE^ Cxcr4^+/F^ mice [52] to obtain conditional ΔT mice (ROSA^ET2-CRE^ CXCR4-ΔT/F) and littermate controls (CXCR4-ΔT/F). To express ΔT specifically in B-lineage cells, ΔT mice were bred to CD19-Cre Cxcr4^+/F^ mice to generate CD19-Cre+ CXCR4-ΔT/F mice. Previously reported CXCR4-null mice were used as negative controls.

### Abelson virus transformation of pro-B cells

FL cells were isolated from E15.5 WT and ΔT embryos and incubated with Abelson virus suspensions (gift from Dr. Hua Gu, Columbia University) for 2 h, 37°C. Cells were then cultured in OptiMEM + 10% FBS. 7 d later, cells underwent a second round of Abelson virus infection. CXCR4-null pro-B cell lines were generated by infecting splenocytes of CXCR4^f/f^CD19-Cre mice with Abelson virus. Primary transformants were analyzed by flow cytometry and confirmed to be a homogenous population of B220^+^CD43^+^ pro-B cells.

### Histology

Histology analysis was performed as described [4]. Briefly, brains of E18.5 embryos were dissected, fixed in Bouin's solution and embedded in paraffin. 6 µm brain sections were stained with hematoxylin and eosin and analyzed under a Nikon Eclipse TE2000-E microscope equipped with a Nikon DS-Fi1 color camera.

### Flow cytometry and receptor internalization

Cells from the PB, spleen or FL were stained using following antibodies: anti-B220 APC, anti-CD43 FITC, anti-CD19 PE, anti-Gr-1 PE, anti-CD11b FITC and anti-IgM FITC (all from eBioscience). Data were attained on an LSR II (Becton Dickinson) and analyzed using FlowJo software (Tree Star).

For receptor internalization analysis, cells were resuspended at 4×10^6^ cells/ml in DMEM and stimulated with 0.5 µg/ml CXCL12 at 37°C. Reactions were terminated by addition of 4% PFA. Cells were stained for surface CXCR4 with anti-CXCR4 biotin (BD) and streptavidin-conjugated PE-Cy7 (eBioscience). CXCR4 downmodulation was determined based on the mean fluorescent intensity (MFI) of CXCR4 expression with the following formula: % downmodulation  = 100 x {(MFI of unstimulated cells (at time t) – MFI of stimulated cells (at time t))/MFI of unstimulated cells (at time 0)}.

### Chemotaxis and adhesion assays

Chemotaxis was performed in transwells (5 µM pore size, Costar). The upper chambers containing 2×10^5^ cells were placed into lower wells with 0.5 µg/ml CXCL12 (Peprotech). After 3 h incubation at 37°C, migrated cells were collected and enumerated by flow cytometry.

Adhesion assays were done as previously described [53]. Briefly, 96-well plates (Greiner) were coated with 1 µg/ml rmVCAM-1-Fc (R&D Systems) and CXCL12 (0.5 µg/ml). Wells were blocked with PBS +2% BSA and 50 µg/ml heparin sulphate. Pro-B cells were seeded at 3×10^5^ cells/well and incubated at 37°C, 2 min. Non-adherent cells were removed by washing and adherent cells counted by flow cytometry.

### Calcium mobilization

5×10^5^ FL cells from E18.5 embryos in 0.5 ml of assay buffer (25 mM HEPES, pH 7.4, 0.1% BSA, 2.5 mM probenecid in Hank's buffer) were loaded for 30 min at 37°C with 2 µM Fluo-4 and 0.84 µM Fura-red (Molecular Probes). Cells were then washed and resuspended in pre-warmed assay buffer. Samples were first analyzed by flow cytometry for 1 min to establish a baseline before stimulation with 0.5 µg/ml CXCL12 for 2 min and then 2 µM ionomycin (Sigma) for another 2 min.

### Immunoprecipitation and immunoblotting

To detect interaction between CXCR4 and G-proteins, WT, CXCR4-null and ΔT pro-B cells were serum-starved for 2 h at 37°C and then cross-linked with 1 mM DSP (dithio-bis-succinimidyl propionate, Pierce) for 30 min, RT. Then cells were lysed by addition of lysis buffer (0.1 M NaCl, 50 mM Tris-HCl, pH 7.4, 15 mM EGTA, 1% SDS, 1% Triton X-100, 1% Cymal-5, 50 µg/ml DNase I, and protease inhibitors (Roche)). Lysates were centrifuged and cleared samples incubated with 10 µg/ml anti-CXCR4 (Abcam) overnight, 4°C, and then with 30 µl protein G agarose for an additional 3 h. Beads were washed, eluted in 2x SDS sample buffer, resolved by 4–12% gradient SDS-PAGE, and analyzed by immunoblotting with anti-Gαi (Santa Cruz Biotechnology).

For detection of pERK and pPKB, splenocytes from conditional ΔT mice and littermate controls were stimulated with 0.5 µg/ml CXCL12 for indicated times and immunoblots prepared from lysates. Western blot analysis was carried out with antibodies against pPKB (S473, Cell Signaling), ERK and pERK (Thr202, Tyr204) (Santa Cruz Biotechnology).

### Radioligand binding assay

HEK cells stably transfected with WT CXCR4 or ΔT were used in a standard competitive radioligand binding assay. Briefly, cells were incubated for 2 hr at 4°C with 187 pM ^125^I-CXCL12 (PerkinElmer) in the presence of increasing concentrations of cold CXCL12 (0–1000 nM). After washing 3 times in wash buffer (0.5 M NaCl, 1% BSA, 5 mM MgCl_2_, 1 mM CaCl_2_, 25 mM HEPES, pH 7.1), bound radioactivity was measured using a β counter (Beckman LS 6500SC). Data were averaged from three independent experiments, and calculated as: % of binding  = 100 x {(cpm_non-comp._ – cpm_sample_)/cpm_non-comp._}

### Intracellular cAMP assay

Splenocytes isolated from conditional ΔT mice and controls were starved in serum-free medium for 1 h at 37°C. After incubation with 1 mM 3-isobutyl-1-methylxanthine (IBMX) at 37°C for 10 min, cells were stimulated with 1 µg/ml CXCL12 for 10 min, followed by 10 min incubation in the presence of 1 µM forskolin. Intracellular cAMP levels were determined using a competitive enzyme immunoassay (Amersham) following the manufacturer's instructions.

### Tango assay

Plasmids β-arrestin-TEV and VTT (TEV cleavage site-tTA fusion construct) were kindly provided by the Richard Axel lab (Columbia University). Luciferase reporter vector (pBI-GI) was from Invitrogen. WT CXCR4 and ΔT were obtained by PCR using primers outlined in *Text S1*, and cloned into the VTT plasmid to generate WT or ΔT fused to the TEV cleavage site ENLYFQL followed by tTA (plasmids WTCXCR4-tTA or ΔTCXCR4-tTA).

HEK cells were plated into a 96-well flat-bottomed plate at 50,000 cells/well and transfected with β-arrestin-TEV, WTCXCR4-tTA or ΔTCXCR4-tTA, and pBI-GI at a ratio of 1∶5∶1 and incubated overnight at 37°C. Supernatant was then replaced with fresh DMEM ±1 µg/ml CXCL12 and incubated for 6 hr, 37°C. Media was aspirated and BrightGlo luciferase substrate (Promega) added to each well and activity measured by a Berthold TriStar LB941 plate reader.

## Supporting Information

Figure S1 **Generation of Mutant Mice.** (A) Structure of the wildtype *Cxcr4* locus, map of the targeting construct, the targeted *Cxcr4* allele before and after Cre/loxP-mediated *neo* gene deletion. The tail-truncation mutation was introduced into the second exon by PCR (Exon II). (B) Southern blot analysis of tail DNA from wildtype, heterozygous mice carrying both the mutated exon II and the *neo* gene (+/−), and heterozygous mice carrying mutant *Cxcr4* allele after *neo* gene deletion (+/mu). The allele detected by probe a: wildtype or mutant *Cxcr4* allele without the *neo* gene: 3.8 kb; the mutant *Cxcr4* allele with the *neo* gene: 5.0 kb. The allele detected by probe b: wildtype: 7.2 kb; the mutant *Cxcr4* allele with or without the *neo* gene: 4.9 kb. The following PCR primers are used for genotyping, which produces a 405-bp wildtype band and a 303-bp tail-truncated *Cxcr4* band: 005-Δ5′: 5′-CTTCTTCCACTGTTGCCTGAACC-3′ 005-Δ3-end: 5′- GCCACAGGTCCCTGCCTAGAC-3′ (TIF)

Figure S2 **B cell precursors in the spleen and peripheral blood.** Splenocytes and peripheral blood cells were isolated from 6-week-old wildtype (ΔT/F), CD19-Cre+ CXCR4^F/ΔT^ (ΔT/-) and CD19-Cre+ CXCR4^F/F^ (−/−) mice and stained with antibodies against CD19, B220 and IgM. CD19^+^-gated cells were plotted with IgM and B220. Numbers (mean ± SD) represent percentages of B220^lo^IgM^−^ B-cell precursors within the CD19^+^ B-cell population (n = 5–8). (TIF)

Figure S3 **Expression of CXCR4, CXCR7 and CD29 in pro-B cells.** (A) Cell surface expression of VLA-4 (α4β1) on Abelson-transformed pro-B cells derived from the wildtype (shaded) and CXCR4-ΔT mice (red line) were detected with anti-CD29 (integrin β1) and analyzed by flow cytometry. (B) Cell surface expression of CXCR4 on Abelson-transformed pro-B cells derived from wildtype, CXCR4-null and CXCR4-ΔT mice. (C) Expression of CXCR7 was determined by qRT-PCR. RNA was purified from fetal liver cells of E18.5 wildtype (WT) or CXCR7^−/−^ embryos, bone marrow cells of wildtype 8-week-old mice, or Abelson-transformed WT pro B cells using an RNeasy kit (Qiagen). cDNA was then prepared using SuperScriptIII First Strand Synthesis kit (Invitrogen). qRT-PCR was performed using primers specific for CXCR7. Each value was normalized to β-actin expression levels. (TIF)

Figure S4 **Pro-B cell adhesion to VCAM-1 is insensitive to pertussis toxin treatment.** Pro-B cells were treated with 100 ng/ml of pertussis toxin (PTX) for 3 h at 37°C. PTX-treated and untreated cells were respectively seeded in 96-well plates coated with 1 µg/ml of mouse VCAM-1-Fc with or without 0.5 µg/ml of CXCL12, and incubated at 37°C for an additional 2 min. Non-adherent cells were then removed by washing, adherent cells were collected and counted. Bar graphs represent the percentages of pro-B cells attached to VCAM-1 in the presence or absence of PTX. Wildtype and CXCR4-ΔT cells are indicated by open and gray bars, respectively. (TIF)

Figure S5 **Impaired signaling in CXCR4 CXCR4-ΔT pro-B cells.** Abelson-transformed pro-B cells were stimulated with 0.5 µg/ml CXCL12 for indicated times. Cell lysates were prepared and Western blot analysis for phosphorylated PKB at serine 473 (pPKB) or phosphorylated ERK (pERK) were performed. Equal loading of protein was confirmed by re-blotting with anti-ERK antibody. Results are representative of at least 3 independent experiments. (TIF)

Text S1 Primers. (DOC)
