# Supplementary figures and images for: An Essential Role of the Cytoplasmic Tail of CXCR4 in G-Protein Signaling and Organogenesis
Source: PLoS One. 2010 Nov 19;5(11):e15397. doi: 10.1371/journal.pone.0015397 (PMC2988825; doi:10.1371/journal.pone.0015397)

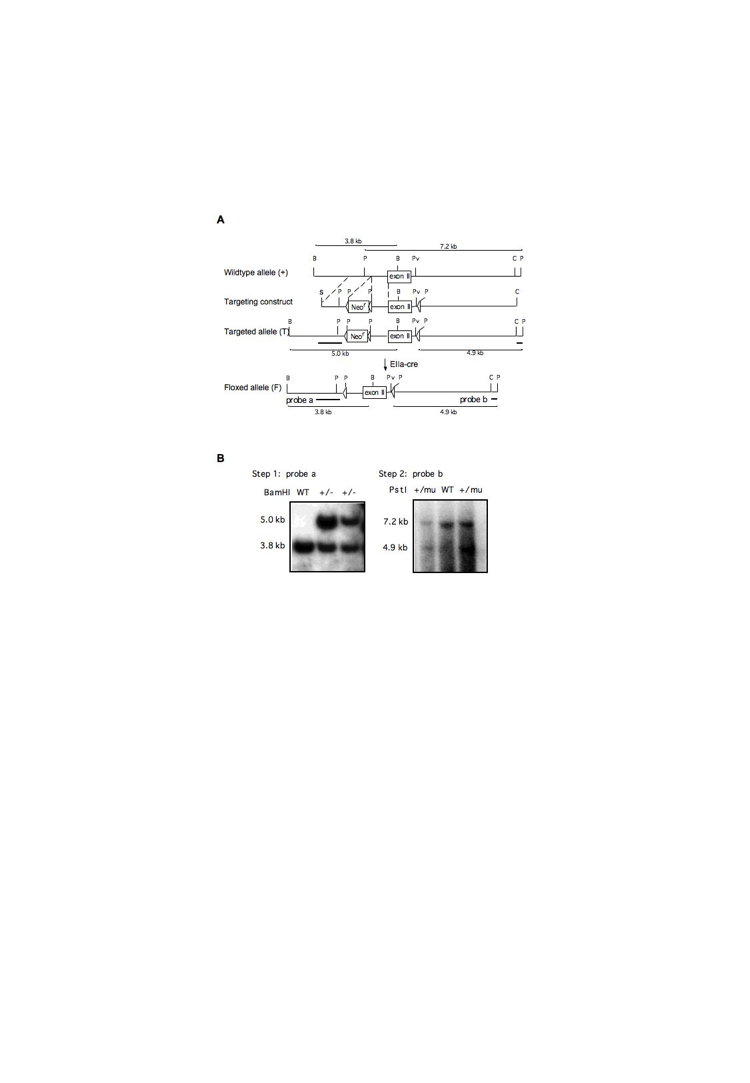

Supplement: Figure S1 — Generation of Mutant Mice. (A) Structure of the wildtype Cxcr4 locus, map of the targeting construct, the targeted Cxcr4 allele before and after Cre/loxP-mediated neo gene deletion. The tail-truncation mutation was introduced into the second exon by PCR (Exon II). (B) Southern blot analysis of tail DNA from wildtype, heterozygous mice carrying both the mutated exon II and the neo gene (+/−), and heterozygous mice carrying mutant Cxcr4 allele after neo gene deletion (+/mu). The allele detected by probe a: wildtype or mutant Cxcr4 allele without the neo gene: 3.8 kb; the mutant Cxcr4 allele with the neo gene: 5.0 kb. The allele detected by probe b: wildtype: 7.2 kb; the mutant Cxcr4 allele with or without the neo gene: 4.9 kb. The following PCR primers are used for genotyping, which produces a 405-bp wildtype band and a 303-bp tail-truncated Cxcr4 band: 005-Δ5′: 5′-CTTCTTCCACTGTTGCCTGAACC-3′ 005-Δ3-end: 5′- GCCACAGGTCCCTGCCTAGAC-3′ (TIF) [file pone.0015397.s001.tif]

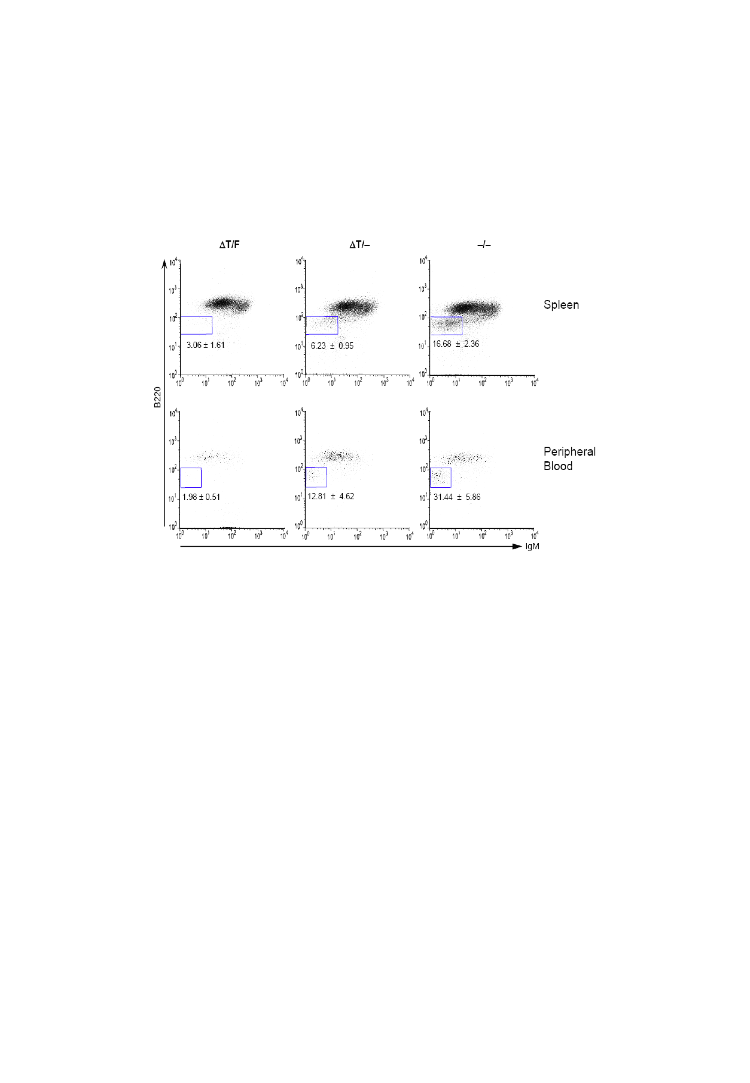

Supplement: Figure S2 — B cell precursors in the spleen and peripheral blood. Splenocytes and peripheral blood cells were isolated from 6-week-old wildtype (ΔT/F), CD19-Cre+ CXCR4F/ΔT (ΔT/-) and CD19-Cre+ CXCR4F/F (−/−) mice and stained with antibodies against CD19, B220 and IgM. CD19+-gated cells were plotted with IgM and B220. Numbers (mean ± SD) represent percentages of B220loIgM− B-cell precursors within the CD19+ B-cell population (n = 5–8). (TIF) [file pone.0015397.s002.tif]

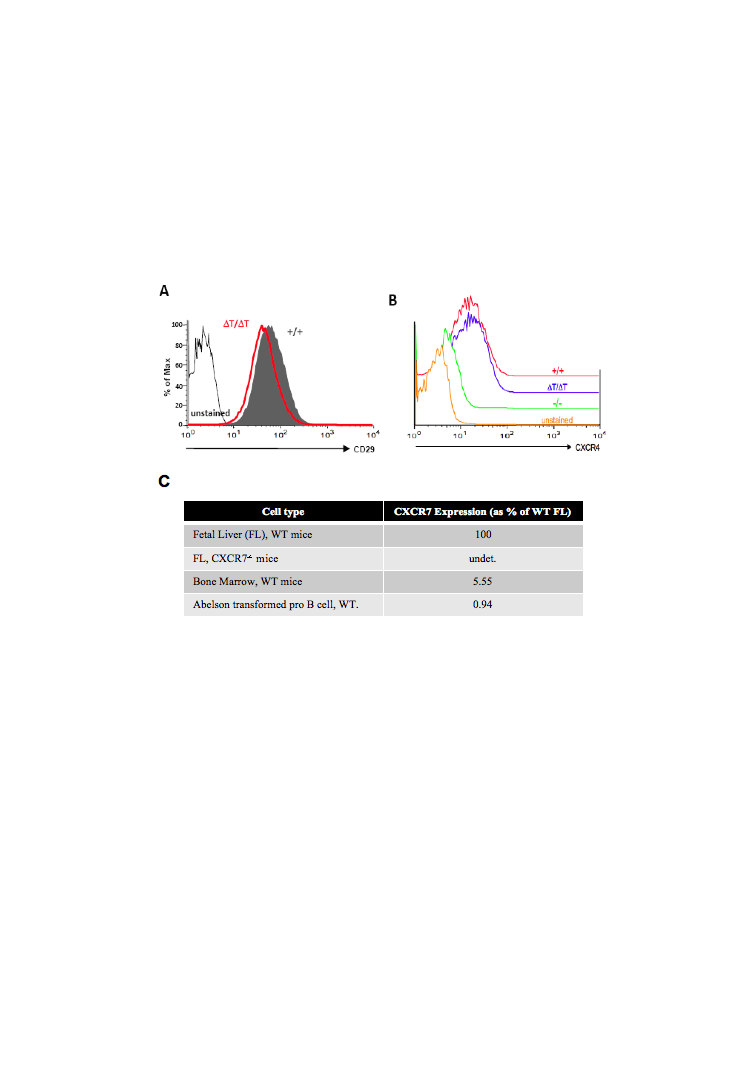

Supplement: Figure S3 — Expression of CXCR4, CXCR7 and CD29 in pro-B cells. (A) Cell surface expression of VLA-4 (α4β1) on Abelson-transformed pro-B cells derived from the wildtype (shaded) and CXCR4-ΔT mice (red line) were detected with anti-CD29 (integrin β1) and analyzed by flow cytometry. (B) Cell surface expression of CXCR4 on Abelson-transformed pro-B cells derived from wildtype, CXCR4-null and CXCR4-ΔT mice. (C) Expression of CXCR7 was determined by qRT-PCR. RNA was purified from fetal liver cells of E18.5 wildtype (WT) or CXCR7−/− embryos, bone marrow cells of wildtype 8-week-old mice, or Abelson-transformed WT pro B cells using an RNeasy kit (Qiagen). cDNA was then prepared using SuperScriptIII First Strand Synthesis kit (Invitrogen). qRT-PCR was performed using primers specific for CXCR7. Each value was normalized to β-actin expression levels. (TIF) [file pone.0015397.s003.tif]

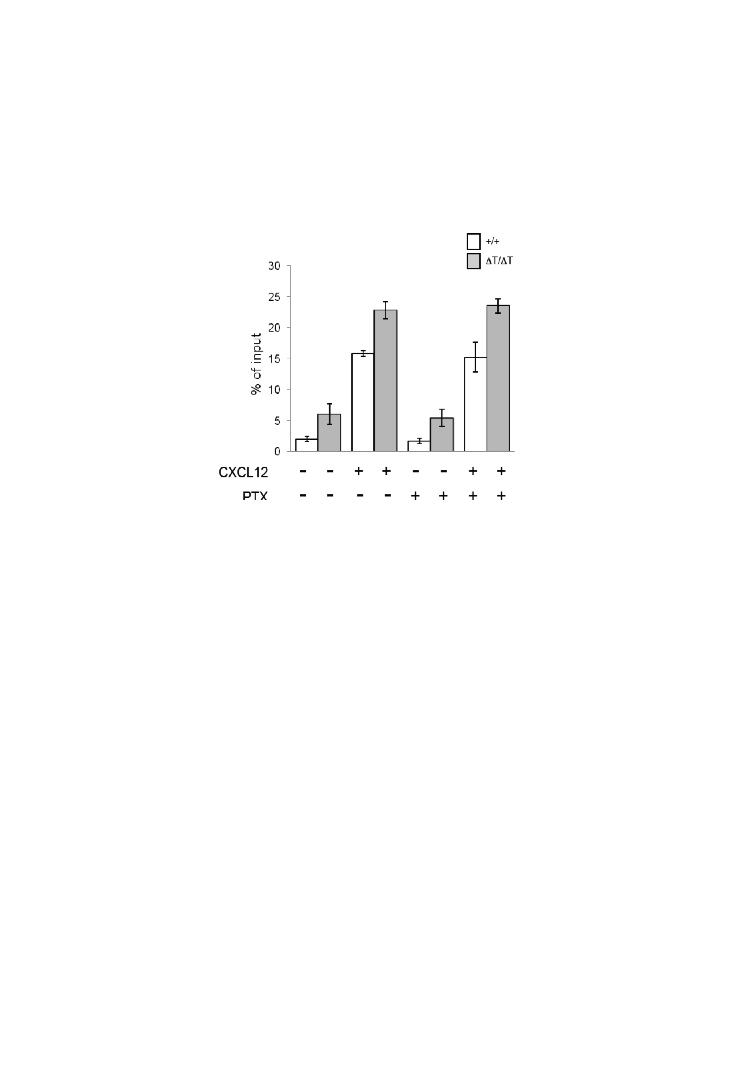

Supplement: Figure S4 — Pro-B cell adhesion to VCAM-1 is insensitive to pertussis toxin treatment. Pro-B cells were treated with 100 ng/ml of pertussis toxin (PTX) for 3 h at 37°C. PTX-treated and untreated cells were respectively seeded in 96-well plates coated with 1 µg/ml of mouse VCAM-1-Fc with or without 0.5 µg/ml of CXCL12, and incubated at 37°C for an additional 2 min. Non-adherent cells were then removed by washing, adherent cells were collected and counted. Bar graphs represent the percentages of pro-B cells attached to VCAM-1 in the presence or absence of PTX. Wildtype and CXCR4-ΔT cells are indicated by open and gray bars, respectively. (TIF) [file pone.0015397.s004.tif]

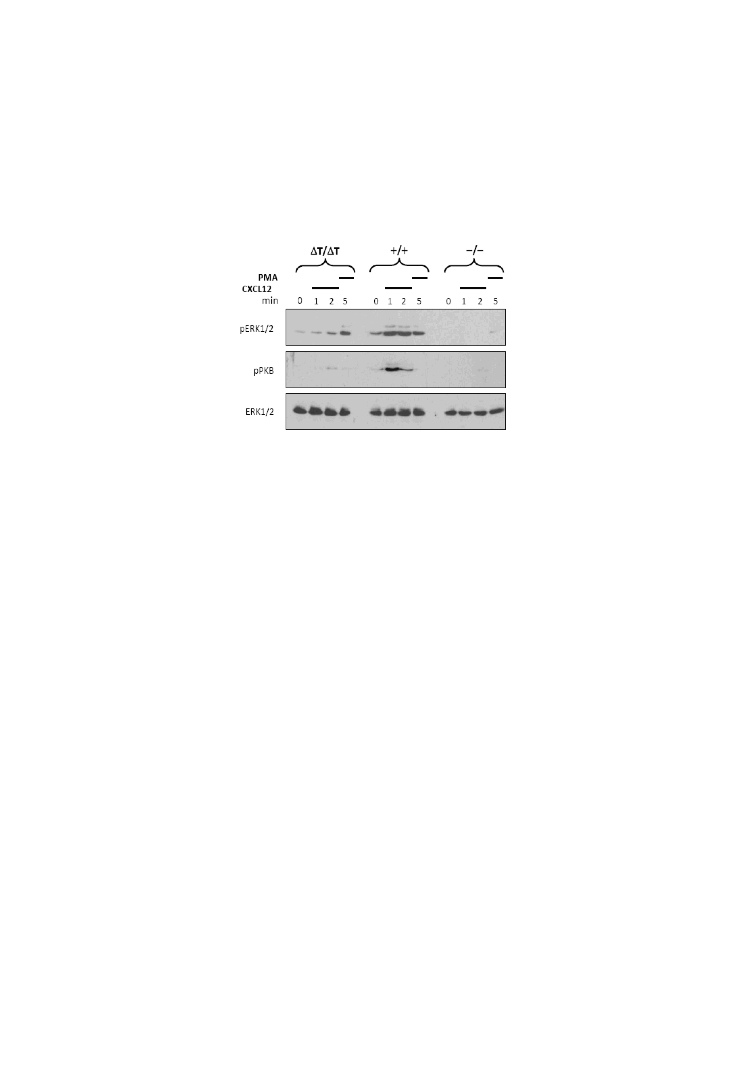

Supplement: Figure S5 — Impaired signaling in CXCR4 CXCR4-ΔT pro-B cells. Abelson-transformed pro-B cells were stimulated with 0.5 µg/ml CXCL12 for indicated times. Cell lysates were prepared and Western blot analysis for phosphorylated PKB at serine 473 (pPKB) or phosphorylated ERK (pERK) were performed. Equal loading of protein was confirmed by re-blotting with anti-ERK antibody. Results are representative of at least 3 independent experiments. (TIF) [file pone.0015397.s005.tif]
