# Supplementary material for: An Essential Role of the Cytoplasmic Tail of CXCR4 in G-Protein Signaling and Organogenesis
Source: PLoS One. 2010 Nov 19;5(11):e15397. doi: 10.1371/journal.pone.0015397 (PMC2988825; doi:10.1371/journal.pone.0015397)
Supplement: Text S1 — Primers. (DOC) [file pone.0015397.s006.doc]

**Supporting Information Text**

**Text S1.** The following primers were used to clone CXCR4 and ΔT before restriction digest and ligation into the VTT vector. CXCR4: *EcoPrimer1: 5’-CGGAATTCAGTGTTGCCATGGAACCGATCAG-3’* and *Bglprimer4: 5’-GAAGATCTGCTGGAGTGAAAACTGGAGG-3’*; for CXCR4-ΔT: *EcoPrimer1* and *BglZC2: 5’-GAAGATCTTTTGAACTTGGCCCC-3’*.
